# Dr. A. M. Straight

**Published:** 1917-03

**Authors:** 


**Dr. A. M. Straight,** Cleveland Medical College (Western Reserve) 1870, Bellevue 1874, of Bradford, Pa., died at his residence in Barondale, a suburb of Bradford, Feb. 19, aged 70.

